# Fibrinogen, Fibrinogen-like 1 and Fibrinogen-like 2 Proteins, and Their Effects

**DOI:** 10.3390/biomedicines10071712

**Published:** 2022-07-15

**Authors:** Nurul H. Sulimai, Jason Brown, David Lominadze

**Affiliations:** 1 Departments of Surgery, University of South Florida Morsani College of Medicine, Tampa, FL 33612, USA; nurulsulimai@usf.edu (N.H.S.); jasonb3@usf.edu (J.B.); 2 Departments of Molecular Pharmacology and Physiology, University of South Florida Morsani College of Medicine, Tampa, FL 33612, USA

**Keywords:** fibrin, fibrinogen chains, fibrinogen derivatives, inflammatory diseases

## Abstract

Fibrinogen (Fg) and its derivatives play a considerable role in many diseases. For example, increased levels of Fg have been found in many inflammatory diseases, such as Alzheimer’s disease, multiple sclerosis, traumatic brain injury, rheumatoid arthritis, systemic lupus erythematosus, and cancer. Although associations of Fg, Fg chains, and its derivatives with various diseases have been established, their specific effects and the mechanisms of actions involved are still unclear. The present review is the first attempt to discuss the role of Fg, Fg chains, its derivatives, and other members of Fg family proteins, such as Fg-like protein 1 and 2, in inflammatory diseases and their effects in immunomodulation.

## 1. Introduction

Fibrinogen (Fg) plays an integral role in blood clotting, and it is a vital protein for survival [1]. However, when its normal content in blood changes, it may lead to various pathological alterations. Inflammation causes an increase in the blood content of Fg and other acute-phase reactant plasma proteins [2,3]. A high concentration of Fg in the blood, called hyperfibrinogenemia (HFg), has been found in many inflammation-associated diseases such as cancer [4,5], rheumatoid arthritis [6], systemic lupus erythematosus [7], Alzheimer’s disease (AD) [8], traumatic brain injury (TBI) [9,10,11], and vascular diseases [12,13] including heart disease [12,14], stroke [15], hypertension [16,17,18], and diabetes [12,19]. There are also disorders associated with alterations of Fg’s function that could be inherited or acquired and can be manifested as altered hemorrhagic and thrombotic susceptibilities. Two examples are afibrinogenemia, a very rare condition characterized by the absence of circulating Fg, and hypofibrinogenemia, which is characterized by a reduced level of circulating Fg. Acquired Fg disorders could be caused by a liver disease that affects the synthesis of normal level of functioning Fg or by a consumptive coagulopathy condition such as disseminated intravascular coagulation [20]. The goal of this review is to provide an overview of some possible mechanisms involved in various inflammatory conditions affected by changes in level of Fg and its derivatives.

## 2. Biosynthesis of Fg, Fibrinogen-like 1 (FGL1), and Fibrinogen-like 2 (FGL2) Proteins

Fg is primarily synthesized in liver hepatic parenchymal cells [21]. Fg consists of three pairs of polypeptide chains, Aα, Bβ and γ, joined by disulfide bonds to form a symmetric dimeric structure [21,22,23]. The assembly of the final form of Fg takes place intracellularly in the endoplasmic reticulum (ER) and requires well-coordinated steps that happen rapidly, within less than five minutes [21]. The intracellular oligomers of Fg chains are the α chain (FGA), β chain (FGB), and γ chain (FGG) with the apparent molecular masses of 66,000, 52,000 and 46,500 Da, respectively [22]. The mechanism of Fg assembly has been well studied and it has been widely accepted that it involves several coordinated steps [22], which include translation of each of the chains, their translocation into the lumen of the ER, and interactions of these chains with nascent proteins that assist in the assembly and folding processes [21]. Briefly, first, the single chains of Fg interact with each other to form Aα-γ and Bβ-γ complexes [21]. Then, the two-chain complexes acquire another chain to form three-chain half-molecules (Aα, Bβ, γ)_1_. Finally, two half-molecules are joined at their N-termini to form a six-chain dimeric hexamer (Aα, Bβ, γ)_2_ [21]. When fully assembled, Fg is secreted into the circulation at the normal concentration ranging from 2 to 3 g/L of plasma. Chaperone proteins play an important role that assist in protein folding, chain assembly, disulfide bond formation, and ER quality control mechanism to ensure that only correctly assembled and folded proteins exit the ER [21,24]. Early on, nascent Fg chains were found being associated with a resident ER chaperone, BiP (GRP78) [25]. The role of chaperone proteins in Fg assembly has been clarified further by Tamura et al., proposing a slightly different Fg assembly process in a two-step manner [24]. The first step is the integration of the β chain into the pre-formed αγ complex to form the trimer. The second is the integration of the two trimers to assemble the hexamer [24]. The proposed two-step assembly process involved participation of ER chaperone proteins Calnexin (CNX), an ER-resident type I membrane protein, and its soluble homologue, calreticulin (CRT) [24]. CNX and CRT are lectin-type chaperones that assist immature protein folding by recruiting endoplasmic reticulum protein 57 (ERp57). It was proposed that CNX temporarily holds the preexisting Fg αγ complex through monoglucosylated N-linked glycans until the newly synthesized Fg β chain is integrated into the αγ complex to form a Fg trimer [24]. Then, this trimer is handed off to ERp57 from CNX. Next, the protein disulfide isomerase ERp57 facilitates the integration of the two trimers into the hexamer by catalyzing the disulfide bonds formation of glycoproteins [26]. Subsequently, the properly assembled Fg hexamer is moved forward to the secretory pathway [24].

Not all the Fg chains that are synthesized are used in assembly of a fully functional Fg protein. Studies that were carried out to test Fg biosynthesis in human hepatocellular carcinoma (HepG2) cells in vitro showed that under normal conditions, when Fg is expressed at basal levels, there is a surplus of Aα and γ chains that form a steady state pool presented as Aα-γ and free γ chains [21]. The surplus Fg chains that are not secreted are eventually degraded by proteolytic lysosomes and proteasomes [21,27]. In addition, it has been shown that there is a quality control mechanism in Fg secretion, that is tightly regulated, allowing retention of certain chains or unfinished complexes and prevent them from being secreted [28]. Thus, only the fully assembled Fg is secreted into the blood circulation [21].

In a study that used Fg knock out transgenic mice, the deletion of Aα chains of Fg not only resulted in elimination of the Aα chain gene product, but also in the secondary elimination of the Fg Bβ and γ polypeptide chains from the circulation [29]. None of the Fg chains were detected in the whole blood samples from the homozygous Aα^−/−^ mice despite the detection of Bβ and γ polypeptide mRNAs in the liver samples. These findings imply that Bβ and γ are synthesized by the Aα^−/−^ mice hepatocytes, but are not secreted into the circulation in the absence of the Aα chain suggesting that only fully assembled Fg can be secreted to the circulation [29]. HepG2 was also shown to retain and accumulate Aα-γ complexes and some free γ chains [21,30].

Besides being primarily synthesized in hepatocytes, Fg synthesis has been reported in fibroblast-like cell lines derived from monkey kidney (COS cells) [25], baby hamster kidney fibroblast cells [31], lung epithelial cells [32], and human breast cancer epithelial cells [33]. The secretion of complete Fg protein from COS cells into the media occurred after co-transfection of these cells with expression plasmids that contained cDNA sequences of the three Fg chains [25]. This implies that Fg assembly does not seem to necessarily be hepatocyte-specific, and a general secretory mechanism might be sufficient to direct the assembly of Fg protein [22].

Although it has been shown that there are extrahepatic routes of syntheses of Fg chains, it remains unclear if these happen only during some pathologies or during normal conditions. Haidaris et al. have shown that the lung epithelial cells were able to synthesize and secrete Fg when stimulated with interleukin-6 (IL-6) and dexamethasone [32]. In another study that shows extrahepatic Fg synthesis, human breast cancer epithelial cells produced low level of intact Fg along with an abundance of Fg intermediate complexes or degraded Aα, Bβ, and γ chain polypeptides [33]. However, this was from an immortal cell line of breast cancer cells and may not be representative of normal cell function. The expression of Fg γ chain mRNA has been shown in the brain [34]. Later it has been shown that human cultured astrocytes and neurons constitutively express all three Fg chains [35]. The same group documented that Fg chains were detected in the brain sections from mice that experienced a subarachnoid hemorrhage and in human brain sections with surgically resected malignant gliomas [35]. In this study, there was more γ chain expression compared to Bβ and Aα chain expression [35]. Interestingly, it has been described that astrocyte engulf Fg/fibrin [36]. Thus, it is possible that, in living organisms, in addition to expressing Fg chains, cells such as astrocytes may contain endocytosed Fg/fibrin protein or its chains. We found that the functional outcome of a Fg and astrocyte interaction results in the death of astrocytes [37].

FGL1, or hepassocin, is a liver-specific 68 kDa molecular weight protein secreted primarily by hepatocytes [38]. It contains a Fg domain at its C-terminal (similar to those in FGB and FGG), which makes it highly homologous to Fg. However, what makes it different from Fg is that it is missing three functional domains: the platelet binding site, the cross-linking region, and the thrombin sensitive site [39]. This makes it irrelevant to coagulation-related functions.

FGL2, also known as fibroleukin, is identified as two distinct isoforms, membrane associated FGL2 (mFGL2) and soluble FGL2 (sFGL2) [40]. mFGL2 is a 70 kDa transmembrane protein expressed in ECs, epithelial cells, dendritic cells, and macrophages [40]. mFGL2 functions as a prothrombinase and is capable of initiating coagulation in tissue by serine protease activity, which can cleave prothrombin into thrombin through a noncanonical pathway [39,40]. sFGL2, which is a 50 kDa protein, is highly expressed by regulatory T cells [39]. It can be secreted into the vasculature and has been found to suppress T cell activation [40]. FGL2 is constitutively expressed in cells of the heart, lung, small bowel, spleen, ovary, uterus, liver, and kidney [41].

## 3. Disorders Associated with Plasma Levels of Fg

Plasma Fg levels are regulated by complex interactions between environmental and genetic factors [42,43]. Based on twin studies it is estimated that only 30–50% of the plasma Fg level is genetically determined [42,43]. Data acquired from a long-term, ongoing cardiovascular cohort, the Framingham Heart Study that began in 1948 (now with its 3rd generation of participants), revealed that Fg is a moderately heritable blood protein that is influenced by gene, environment, and disease status [44]. These rare congenital Fg disorders can be subclassified in type I and type II disorders. Type I disorders (afibrinogenemia and hypofibrinogenemia) reflect level of Fg in blood (amount of Fg < 1.8 g/L), whereas type II (dysfibrinogenemia and hypodysfibrinogenemia) affect primarily the quality of Fg in the circulation [45,46]. According to the European Network of Rare Bleeding Disorders (EN-RBD) along with the International Society of Thrombosis and Hemostasis, Fg deficiency may be classified into mild hypofibrinogenemia (lower limit of normal level—1.0 g/L), moderate hypofibrinogenemia (0.9–0.5 g/L), severe hypofibrinogenemia (0.5–0.1 g/L), and afibrinogenemia (<0.1 g/L) [47,48].

Several inherited and acquired Fg disorders have been described that affect the quantity (afibrinogenemia and hypofibrinogenemia) or the quality/property (dysfibrinogenemia) of circulating Fg that cause abnormalities in the function of the Fg molecule in some cases resulting in noticeable pathologies such as bleeding or slower clotting time [49]. For example, afibrinogenemia, a rare bleeding disorder, is a result of mutations in any of the 3 genes (FGA, FGB and FGG) [50], and most patients are descendent of consanguineous marriages [51] while dysfibrinogenemia are often related to pathogenic variants affecting specific residues in the amino-terminal region of the Fg Aα-chain [52]. There are over 100 mutations throughout the three Fg chain genes, with the most common mutation being a substitution of the arginine residue in the Aα chain with either histidine or cysteine, making up 63% of dysfibrinogenemia cases [53]. The two mutation hotspots are FGA Arg35 and FGG Arg301. These mutations, in exon 2 of FGA and exon 8 of FGG, make up about 85% of all congenital mutations in dysfibrinogenemia [54]. In patients, clinical manifestations of congenital dysfibrinogenemia are highly heterogenous.

The low Fg level is reflected by abnormal clotting times with a tendency to bleeding, which is further exaggerated more in patients with afibrinogenemia than with hypofibrinogenemia [49]. On the other hand, dysfibrinogenemia that is associated with disorder in Fg structure, which can be congenital or acquired in origin may or may not result in abnormal function [55]. In fact, some experts believe that afibrinogenemia should be considered as a subset of dysfibrinogenemia since several of the afibrinogenemia condition are shown to result from structural defects [55]. Regardless of the classification or nomenclature, bleeding in patients with Fg disorders can vary from asymptomatic, to mild, to severe [49]. A Fg-deficient mice model with a homozygous αA chain-deficiency are born normal in appearance and there is no evidence of fetal loss of these animals based on Mendelian pattern of transmission of the mutant Aα chain allele [29]. Despite that the blood samples from Aα chain-deficient homozygous (Aα^−/−^) mice fail to clot or support platelet aggregation in vitro, remarkably, most newborns displaying signs of bleeding, ultimately control the loss of blood, and survive the neonatal period [29]. Approximately 30% of the Aα^−/−^ newborns mice develop overt bleeding and out of this, 2/3 of them survive the neonatal period. Out of those who survive, 90% survive until weaning and only half survive beyond 70 days [29]. There is also a hereditary hypofibrinogenemia with hepatic storage (HHHS) disorder that involves inborn errors of metabolism involving secretory proteins [49,56]. The HHHS is characterized by hepatocellular storage in the rough ER and a plasma deficiency of relevant proteins such as α-1-antitrypsin, Fg, and α-1-antichymotrypsin [49,56]. The patients suffering from HHHS risk the clinical manifestation associated with coagulopathy due to the hypofibrinogenemia and may also suffer from liver disease due to the protein accumulation in the ER of hepatocytes that resulting in various severity of liver disease [49,56]. Excluding its genetic etiology, Fg disorders are more commonly found in consumption coagulopathy due to liver disease or disseminated intravascular coagulation (DIC) that could lead to hypofibrinogenemia and/or dysfibrinogenemia [57]. In the light of the severe acute respiratory syndrome coronavirus 2 (SARS-CoV-2), aside from its main clinical complication in the pulmonary system, DIC is shown to be one of the major underlying causes of death in afflicted patients [57]. These patients show signs of consumption coagulopathy with the DIC, a decrease in the blood level of Fg, and an increase in the concentration of fibrin degradation product, D-dimer [57]. The patients with afibrinogenemia, hypofibrinogenemia, dysfibrinogenemic, and consumption coagulopathy including in DIC, characterized by the functional impairment of Fg described earlier, not only face the risk of bleeding events but paradoxically risk developing of thrombosis [49,57,58]. Due to Fg role in both the procoagulant and fibrinolytic pathways, the defects in Fg function might be associated with increased risk for hemorrhagic and thrombotic disorder [55]. Managing thrombosis in these patients is challenging as anticoagulant treatment may exacerbate the underlying risk of bleeding [58].

During severe trauma, massive bleeding that is associated with a resultant decreased blood level of Fg is quite common. In addition, there are other factors that affect Fg metabolism and reduce its availability in patients with severe trauma. Hypothermia reduces Fg synthesis, acidaemia following hypoperfusion increases the breakdown of Fg, and diluting blood by intravenous infusion of crystalloid fluid or synthetic colloid has been shown to reduce the content of Fg and Fg-fibrin conversion [59]. This is especially critical with patients with genetic disorders such as hypofibrinogenemia or afibrinogenemia. Infusion of a Fg concentrate is often the replacement therapy of choice [50]. In cases of trauma, it is recommended that Fg levels should be raised, initially to 1 g/L and maintained at this level until hemostasis is established and then kept at least above 0.5 g/L until complete wound healing [50].

There is also a genetic disorder that is associated with HFg, a condition comprised of polymorphisms where the β fibrinogen promoter -455A allele has been shown to be associated with a higher (2.8–3.7 g/L) plasma level of Fg and an increased risk of atherothrombotic disease [43]. In another study, a polymorphism of the β fibrinogen with homozygosity of the β-148T allele was shown to be associated with a sixfold increased risk for atherosclerosis of the carotid artery [60]. A clinical study with patients undergoing coronary artery bypass grafting showed that carriers of the -148T allele had higher preoperative plasma Fg and C-reactive protein (CRP) levels compared to those in non-carriers, as well as increased levels of CRP and IL-6 postoperatively [61].

Age is one of the factors that could affect the concentration of Fg in blood. Level of Fg tends to increase with age, and it is known that average plasma concentrations of Fg are higher in females (3.5 g/L) compared to that in males (3.3 g/L) [62,63]. Although it is statistically significant, the clinical significance of this difference is debatable. Interestingly, smokers show higher average plasma concentrations than non-smokers [62] and exposure to traffic pollutants is also associated with higher Fg concentrations [44]. This might be due to particles deposited in the lungs inducing alveolar inflammation. Various factors such as age, obesity, physical activity, and a disease status have been reported to elevate Fg concentration [62]. The influence of diet on the Fg level in plasma is only modest, with patients with high lipid levels having high levels of Fg, however consumption of fish oil and moderate consumption of alcohol are associated with lower Fg level [42].

It has been well documented that Fg levels are positively correlated with metabolic syndrome [62]. Metabolic syndrome is characterized by the presence of abdominal obesity, atherogenic dyslipidemia, raised blood pressure, presence of insulin resistance, and prothrombic and inflammatory states that predispose to cardiovascular diseases [64]. Some of the factors contributing to the pathogenesis of the metabolic syndrome are obesity, sedentary lifestyle coupled with a deleterious diet, and genetic factors [64]. Unfortunately, these risk factors are also common for patients that suffer from chronic obstructive pulmonary disease (COPD) since the physical limitations imposed on patients due to respiratory symptoms places them at risk of adopting a sedentary lifestyle associated with an increased risk for weight gain, insulin resistance, and development of metabolic syndrome [64]. Fg’s level in plasma is found to be positively correlated with the development and severity of COPD, COPD-related hospitalization, and increased risk of the resultant death [65]. As an acute phase protein, Fg level screening in diseases and conditions mentioned above is proven useful as a biomarker for generalized state of inflammation or a marker for polymorbidity that warrants further workup.

Although it is known that the singular chains of Fg do not circulate in blood [21], FGA precursor protein has been found in cerebrospinal fluid (CSF) collected from human patients [66]. The FGA precursor was found at a higher level in patients with Alzheimer’s Disease (AD) compared to that in patients diagnosed with mild cognitive impairment and age-matched normal controls [66]. In the same study, the level of FGA precursor was positively correlated with the severity of cognitive impairment with its highest concentration seen in the group of patients demonstrating severe impairment of memory and cognition [66]. Although it is proposed as a good source of the biomarker (FGA) for the severity of cognitive impairment in AD patients, the procedure to collect CSF samples is too invasive and painful to make it practical. Fg, in an amount ranging from 0.002 to 0.008 g/L was detected in the CSF of patients with brain disorders including bacterial or viral meningitis, Guillain-Barré, and major depressive disorder has been documented [67]. How Fg and the FGA precursor enter the CSF remains unclear. CSF is derived mainly from brain capillaries, and it is in continuity with the interstitial fluid of the brain. Fg levels in the plasma are almost 1000 times higher than its levels in CSF [67]. In that study, the authors imply the possibility of Fg ‘leakage’ from the blood based on their finding that the total protein concentration in the CSF samples was also elevated [67]. Neurons are bathed by the extracellular fluid of the brain which forms the microenvironment of the central nervous system (CNS) [68]. Despite the close proximity, the molecular exchange between the blood and the neural tissues, or the interstitial fluid environment of neurons, are limited and regulated by existing barriers. These barriers include the blood–brain barrier (BBB), mainly formed by the cerebrovascular endothelial cells (ECs) between the blood and the brain interstitial fluid (ISF); the choroid plexus epithelium—between the blood and ventricular CSF; and the arachnoid epithelium between the blood and subarachnoid CSF [68]. The BBB formed by the cerebrovascular ECs between blood and ventricular CSF, and by the arachnoid epithelium between blood and subarachnoid CSF, restricts the exchange between blood and the CNS tissue [68]. The brain cells are cushioned and supported by two forms of brain-specific fluids, which are the brain ISF and CSF. The ISF occupies the brain interstitial system, whereas the CSF fills the brain ventricles and the subarachnoid space [69]. The CSF serves as a reservoir for the ISF and extensive communication between the ISF and the CSF facilitates the removal of waste products from the brain ISF [69,70]. Molecules released from brain cells can easily diffuse into the CSF [67]. This may be a reason that Fg chains produced in astrocytes and neurons [35] have been found in the CSF [66].

Thrombosis can be defined as an increased hemostatic response that results in the formation of an occlusive blood clot and the obstruction of blood flow in vasculature. Whereas inflammation is characterized by the complex protective immune response to harmful stimuli [71]. Cancer is an inflammatory disease where, in most cases, plasma Fg is increased, and the patients have a higher risk of developing thrombosis. In general, this occurs due to increased blood viscosity, the resultant activation of endothelial cells, and platelet thrombogenic properties. The functional interdependence of thrombosis and inflammation are well-recognized [71].

It is known that inflammation is a critical component of tumor progression [72]. Therefore, it is not surprising that high levels of Fg, which is an acute phase reactant protein synthesized during inflammation, are commonly found in cancer patients. Higher level of Fg in blood has been strongly associated with poor prognosis and lower survival rate in most types of cancers including colon [4], gastric [73], cervical [74], renal [75], and liver [76]. Furthermore, HFg could lead to complications in thromboembolic events in cancer patients [77]. Despite the mounting evidence that an increased Fg level can be associated to poorer prognosis [4,73,74,75,76], the expression of the precursor of FGA, based on the cancer genome atlas data, has been shown to be associated with a favorable prognosis and higher patient survival rate in liver cancer [78]. Increased expression of FGA may also be favorable in lung cancer where it was suggested to have a suppressive role inhibiting tumor growth and metastasis in human lung adenocarcinoma [79]. FGA was shown to directly bind integrin α5 on the ECs, the known Fg receptor [80]. Interaction of FGA with the integrin α5 results in reduced phosphorylation of AKT leading to inhibition of mammalian target of rapamycin signaling [79]. It was found that the knockout of FGA promotes tumor growth and metastasis, suggesting the role of FGA as a suppressor for tumor growth and metastasis in the lung cancer [79]. FGA expression was the highest in the hepatocellular carcinoma cell line HepG2, more than in lung cancer cell lines such as A549 and H1299 [79]. Expression of FGA was not found in prostate (LNCaP, PC3 and DU145) and breast cancer cell (MBA-MB-231 and MCF7) lines [79]. The two types of cancers that show the highest expression of FGA are liver and lung cancers [79]. Interestingly, these two cancers are the only types of cancer that are associated with a better prognosis and outlook for the disease when higher level of FGA is detected. 

Activation of the extrinsic coagulation system and the fibrinolytic cascade may be related with growth, invasion, and metastasis of tumor cells [81]. It is indisputable that there is crosstalk between coagulation and inflammation, both being affected by Fg. An inflammatory response shifts the hemostatic system toward a prothrombotic state while coagulation, in parallel, affects inflammation [82]. During coagulation, cleavage of Fg by thrombin results in release of fibrinopeptide A and fibrinopeptide B (FpB) and triggers fibrin polymerization [82]. FpB has been shown to be a potent chemotactic agent for polymorphonuclear neutrophils and fibroblast [83]. Other Fg/fibrin degradation products have also been shown playing a role in the inflammatory reaction. These are, for example, the Fg fragment D, D-dimer generated by plasmin digestion of fibrin that is commonly used as a biomarker for fibrinolysis and DIC, fibrin fragment E, and Bβ15-42, a fragment of the *N*-terminal β chain [82]. It is well documented that digestion of Fg by plasmin in vivo is quite rare [84]. During many inflammatory diseases, increased content of Fg and plasminogen-activator inhibitor type 1 leads to decreased fibrinolysis and an accumulation of Fg [85]. It has been shown that Fg and β-amyloid association, which is a common pathology in inflammatory AD, alters thrombosis and fibrinolysis [86]. Thus, a higher content of Fg and the reduction in fibrinolysis results in accumulation of Fg/fibrin during inflammatory diseases (e.g., during AD and TBI).

Like Fg, FGL1 was found to be increased in an experimental model of acute inflammation [38,39] indicating its role in an acute phase reaction. In that study, IL-6 was used to activate Hep G2 cells, a human hepatocyte carcinoma cell line, which resulted in increased FGL1 expression in vitro [39]. In the same study, subcutaneous injections of turpentine oil in rats were used to stimulate inflammation at an extrahepatic site and resulted in an increased serum level of FGL1, which was dampened by dexamethasone, an IL-6 biosynthesis suppressor [39]. FGL1, which was thought to be an acute phase reactant protein [39], was detected in the non-diseased pancreatic samples from patients with a benign pancreatic condition [5].

FGL2 also has potential to be used as a biomarker for certain condition since circulating sFGL2 was found to be correlated with viral loading and disease severity in patients with human hepatitis B virus or hepatitis C virus (HCV) infections [40,87]. Plasma level of FGL2 was shown to be positively correlated with chronic HCV infection titers and the degree of inflammation in the liver [87]. Among the HCV patients, higher FGL2 level is associated with the severity of fibrosis. In a study with mouse hepatitis virus 3 (MHV3), a member of coronaviridae (large, enveloped, single-stranded RNA viruses), administration of FGL2-mAb resulted in a dose-dependent reduction in the MHV3 viral titers, improved liver histology and the survival rate of patients [88]. Target-specific inhibition of mFGL2 expression may have a potential for use in hepatitis therapy [88] and the immunosuppressive effect of sFGL2 warrants a further exploration.

In the tumor microenvironment, Fg regulates the expression of genes involved in cell cycle regulation and metabolism, promotes tumor growth, and limits tumor cell senescence [89]. During inflammation, the blood level of Fg increases and remains elevated for more than 21 days [2]. In cases of severe TBI, Fg concentration initially dropped due to trauma-induced loss of blood, but its concentration increased above 4 g/L two days after injury, peaked to the level of 5.8 ± 0.35 g/L on day 6, and remain elevated for 2 weeks [10]. It is known that, after head injury, there is an acute BBB disruption not only in severe but also in some of the mild and moderate TBI [9]. It has been shown that in some of the moderate or severe cases of TBI, the BBB disruption persist long after the impact at the site of contusion [9]. We have shown that at high levels Fg altered cultured EC layer integrity through downregulation of vascular endothelial cadherin and matrix metalloproteinase-9 activation [90] along with alteration in the expression of actin-associated endothelial tight junction proteins [91]. An interaction of Fg with endothelial ICAM-1 and α5β1 integrin caused a dose-dependent increase in EC layer permeability to albumin and to the Fg itself [92]. This Fg-induced increased endothelial permeability occurred in conjunction with enhanced formation of F-actin and the formation of gaps in the EC monolayer [92]. However, during neuroinflammation, such as TBI, HFg enhanced formation of functional caveolae in the mouse brain ECs [93] resulting in increased cerebrovascular permeability mainly through caveolar protein transcytosis [94,95]. The increased cerebrovascular permeability associated with HFg that occurred during TBI [11] and with HFg in general [96] can be one of the strongest mechanisms for deposition of Fg in the brain parenchyma in response to inflammation. Fg deposits were found in postmortem brain samples from humans diagnosed with TBI [9,97]. Fg deposition in the brain perivascular space was more diffused in instances of acute TBI [97], but it was less diffused in samples with long term survival, chronic TBI [9,97]. One study shows extensive Fg and IgG immunoreactivity in brain sections from the superior frontal gyrus in a TBI patient who survived 18 years after a fall [9]. Similar to findings in human TBI cases [9,10], we found that mild-to-moderate TBI in mice was accompanied with HFg [11]. Due to increased cerebrovascular permeability Fg was translocated to the extravascular space of the brain [11] where it was deposited in the vasculo-astrocyte endfeet interface [98]. The extravasated Fg formed complexes with proteins such as amyloid beta (Aβ) [96] in addition to astrocytic and neuronal intercellular adhesion protein-1 (ICAM-1) and cellular prion protein (PrP^C^) [11,99,100]. Formation of these protein complexes were associated with a reduction in short-term memory [11,96] indicating a possible cause and effect relationship between the HFg and cognitive impairment during neuroinflammatory diseases [96,101].

HFg is not only a biomarker of inflammation [3], but it has been shown to be a cause of inflammatory responses [11,13,90,91,92]. We have previously shown that Fg dose-dependently activates astrocytes [102] and induces upregulation of ICAM-1, TrkB, cytokines such as C-X-C motif chemokine 10, IL-6, and C-C motif chemokine 2 (CCL2) [37,102]. In neurons, HFg induced upregulation of IL-6, and increased generation of reactive oxygen species, mitochondrial superoxide and nitrite [99]. We found that Fg association with its astrocytic and neuronal receptors ICAM-1 and PrP^C^ [37,99,102,103] are in part responsible for the pro-inflammatory reaction and increased oxidative damages in astrocytes that resulted in apoptosis of neurons [37,99,104]. Our in vivo studies indicate stimulation of activating transcription factor 3 (ATF3), a marker of inflammation, in mouse brain samples 14 days after cortical contusion injury result in mild TBI [104]. This effect of ATF3 overexpression, and thus inflammation, was ameliorated in brains from mice treated with Fg antisense oligonucleotide (inhibits synthesis of Fg) [104]. Fragment D that is generated during fibrinolysis, has been shown to have a potent pro-inflammatory effect [105].

Multiple sclerosis (MS) is a chronic inflammatory autoimmune disorder characterized by lymphocytic infiltration, demyelinating white matter lesions with perivascular inflammation, and axonal damage concentrated in the CNS [106]. HFg is considered a risk factor for patients with MS with plasma levels of Fg exceeding 4.17 g/L and MRI scans demonstrating active lesions [107]. Moreover, it is stipulated that Fg is not only a biomarker for MS, but it is also involved in the development of the disease. The pro-inflammatory role of Fg in the pathology of MS has been shown in the experimental animal model of autoimmune encephalomyelitis [108]. A Fg injection into the cortex of Cx3cr1^GFP/+^ reporter mice for resident monocytes, induced rapid and sustained microglial responses in vivo, was associated with axonal damage and release of reactive oxygen species in microglia [108]. The same group has shown that Fg deposited in the CNS induces encephalitogenic adaptive immunity responses and peripheral macrophage recruitment into the CNS leading to demyelination [109]. Interestingly, inhibition of Fg receptor CD11b/CD18 provided protection from the immune and neuropathologic effects [109]. This suggests that Fg contributes to the pathology of the autoimmune encephalomyelitis in MS.

### Fibrinogen Hindering Autoimmune Cell Reaction

Fg conversion to fibrin is a part of normal hemostasis necessary for wound healing. In fact, Fg-fibrin grafts have been used as dressing to promote hemostasis and slow wound healing during diabetes [110]. Fibrin mesh that forms over the skin wound has been shown to form a barrier and effectively defend against microbial invasion [111]. Interestingly, it has been reported that the exposure of Fg to disulfide-reducing agents results in the formation of insoluble aggregates, which if adhered to tumor cells act as a barrier to tumor recognition by the innate immune system [112]. Similar aggregates could be formed by involvement of free iron [113]. In the absence of specific chaperons, the exposed hydrophobic epitopes of Fg form scrambled linkages forming fibrin-like fibrils (parafibrin). Parafibrin has a total resistance to proteolytic degradation. As a result, once attached to the surfaces of cells, parafibrin induces a permanent state of inflammation resulting in the release of cytokines and proteases from macrophages impairing their functions [114,115]. Destructive effects of parafibrin have been shown in pathogenesis of AD [114]. It has also been shown that fibrin can form a physical barrier that protects the cancer cells from the action of natural killer (NK) cytotoxicity [116]. The study showed that only a limited quantity of NK cells adhered to the tumor cells, suggesting that Fg/fibrin blocked the formation of an effector-target conjugate with approximately up to 80% effectiveness [116].

Clinically apparent tumors develop mechanisms to evade autoimmune elimination. Immunotherapy drugs work by using the body’s immune system to fight cancer. Immune checkpoints on cell surfaces help control the immune responses. Usually, immune checkpoints keep T cells inactive until they are needed to keep the T cells from harming normal cells. Cancer cells can take advantage of these checkpoints as part of their immune evasion mechanism to avoid being killed by autoimmune cells. The immune checkpoint inhibitors work by blocking checkpoint proteins from binding with their partner proteins. This prevents the “off” signal from being sent and allows the T cells to be “on” and to kill cancer cells. One of the most promising immune checkpoints is lymphocyte-activation gene 3 (LAG-3). LAG-3 is expressed on the surface of lymphocytes including CD4^+^ T cells, CD8^+^ T cells, NK cells, and regulatory T (Treg) cells [117]. The identification of FGL1 as a functional ligand to LAG-3, suppressing T cell responses, was an exciting finding in cancer immune checkpoint inhibitors therapy [118]. FGLI is highly produced by human cancer cells [118]. High LAG-3 and FGL1 expression boosts tumor growth by inhibiting the immune microenvironment, essentially helping the tumor cells to evade autoimmune elimination. Blocking the interaction between LAG-3 and FGL1 with a monoclonal antibody increases intertumoral T cell responses and leads to decreased tumor size in murine models of melanoma [118].

Similarly, the soluble form of FGL2, sFGL2, has been shown to acts as an immunosuppressor [40]. FGL2 has been shown to suppress alloreactive T lymphocyte proliferation and the maturation of bone marrow dendritic cells [40,119]. Furthermore, anti FGL2 antibody significantly blocked the suppressive activity of Treg-cells, suggesting its role as an effector of Treg function [120]. Transgenic mice with deletion of FGL2 (*fgl*2^−/−^), spontaneously develop autoimmune glomerulonephritis with an increase in age [121]. Deficiency of FGL2 resulted in increased T cell proliferation [121]. Conversely, when sFGL2 was used to observe its effect on T cell proliferation, it was capable of inhibiting T cell proliferation activated by various stimuli, including alloantigens anti-CD3/anti-CD28 monoclonal antibodies, in a dose dependent manner [122]. This further strengthen the hypothesis that FGL2 exerts immunosuppressive effects on T cell proliferation and dendritic cell maturation [122]. The immunosuppressive effects of FGL1 or FGL2 makes them a good target in a promising immune checkpoint blockade strategy during treatment of cancer, especially because they are upregulated in various solid tumors [38]. The challenge of using a checkpoint inhibitor in general is that each of them has a distinct side effect. They may not only have the desirable anti-tumor immune reactivity but may be accompanied by unintended activation of non-tumor specific immune responses that target self-antigens on healthy tissues. Moreover, not all types of cancer are treatable by this type of immune therapy. In March 2022 the FDA approved relatlimab, the first immunotherapy drug that targets the LAG-3 immune checkpoint pathway. There are 8 other immune checkpoint therapies already approved by the FDA that are targeting the programmed cell death protein 1/programmed death-ligand 1 and cytotoxic T-lymphocyte-associated protein 4 checkpoints. The finding of FGL1 as a functional ligand to LAG-3 opens the door for a greater possibility of utilizing FGL1 [118]. In addition, FGL1 is upregulated in various solid tumors such as lung, prostate, melanoma, colorectal, breast cancer, and brain tumors [38]. Targeting FGL1 is advantageous as it targets the tumor cells and exploit its effect on immune cells, making it a superior selective therapeutic strategy. More studies are necessary to understand mechanisms and effects, which can provide a novel strategy for tumor therapy.

## 4. Fibrinogen Signaling

We found that a Fg interaction with PrP^C^ induced overexpression of tyrosine receptor kinase B (TrkB) and activation of astrocytes [103]. TrkB is a receptor for brain-derived neurotropic factor which is a critical growth factor in neuronal cell growth, differentiation, morphology, and synaptogenesis [123]. This Fg-induced upregulation of TrkB [103] coincided with other data showing that overexpression of TrkB in astrocytes is associated with increased nitric oxide production and nitrotyrosine deposition that ultimately promote neurodegeneration [124]. We have also found that Fg induced upregulation of pro-inflammatory cytokine interleukin 6 not only in astrocytes [37] but also in neurons [99]. Furthermore, Fg induced enhanced generation of reactive oxygen species and nitrite in astrocytes [104] and mitochondrial superoxide in neurons [99]. All these indicated a presence of oxidative damage, which led to apoptosis and increased cell death in astrocytes and neurons [37,99]. These effects, including Fg-induced upregulation of the pro-inflammatory cytokines, oxidative damage, and increased cell death, were ameliorated when the functions of ICAM-1 or PrP^C^ were blocked with respective function blockers [37,99], thus indicating a specific effect of Fg.

Fg has been shown to induce activation of NF-κB in human pancreatic stellate cells (PSC) via binding to α_v_β_3_ and α_5_β_1_ located on the cells [125]. In that study, Fg induced upregulation of IL-6 in the PSC in 24 h, similar to what we found in our study [37,99]. Fg was also found to activate three classes of MAPKs (extracellular signal-regulated kinase (ERK), c-Jun N-terminal kinase (JNK) and p38 MAPK, and Akt) in a time-dependent manner, most prominently between 5 to 60 min after incubation with the PSC cells [125]. The Fg-induced upregulation of IL-6 and interleukin-8 was almost completely inhibited when an NF-κB inhibitor was used, but only partially inhibited by inhibitors of ERK and p38 MAPK [125], suggesting that NF-κB may play a greater role in Fg transduction in the PCS. Although it has never been shown before, it is possible that Fg-induced pathology during TBI involves the NF-κB signaling pathway, which is best known for its regulation of inflammation and innate immunity.

The role of Fg in cerebrovascular dysfunction during TBI has been shown [96], and discussed in detail in our previous reviews [101,126,127]. However, the connecting signaling pathway of Fg-induced pathology during TBI was not clear. Identifying the mechanisms and signaling pathways that govern the contribution of Fg to the pathology during TBI may reveal the window of opportunity for therapeutic target(s) and possible interventions. It has been shown that NF-κb was detected in different type of cells in the brain during TBI including ECs, neurons, astrocytes, and microglia [128]. Interestingly, the temporal distribution of the NF-κB activity was detected in the different type of cells in the brain during TBI, with the shortest duration (up to 1 week) detected in neurons and activity persisting longer (up to 1 year) in the non-neuronal cells [128]. In the same study, little or no NF-κB immunoreactivity was detected in sham-operated rats or in the contralateral hemispheres of the contused animals. In neurons, NF-κB was detected first in axons within 1–2 h post injury and later in the soma (including the nucleus). NF-κB detection was limited to within 1 week in neurons, primarily in the injured cortex, and it was no longer detected in 2 weeks [128]. NF-κB was detected in morphologically reactive astrocytes, but not in normal astrocyte [128]. The NF-κB immunoreactivity detected in reactive astrocytes was found 48 h after injury, with the most intense immunoreactivity found 1 week after the injury, and it lasted up to 1 year [128]. In microglia, the NF-κB was detected from 24 h to 1 year after injury with most pronounced effects found from 48 h to 1 week post injury. In vascular ECs, NF-κB was detected early, 1 h after injury, and its expression persisted up to 1 year [128]. The long-lasting NF-κB activation during TBI might contribute to the prolonged inflammation and neurodegeneration seen in the delayed pathology of TBI. The specific source of persistent post traumatic activation of NF-κB in the study was not clear. Identifying the initiating culprit and the pathogenesis of how it contributes to the NF-κB activation may reveal the window of opportunity for therapeutic target(s).

We have previously shown that HFg induced ERK phosphorylation in rat cardiac microvascular ECs [92]. Fg dose-dependently increased F-actin formation, ERK phosphorylation, and albumin leakage through the EC monolayer, which were reduced when mitogen-activated protein kinase (MEK)/ERK inhibitors were used [92].

It has been shown that Fg in the extracellular matrix of colorectal tumor cells interacts with integrin receptors that trigger the downstream effector molecule, focal adhesion kinase (FAK), on the cytosolic segment of focal adhesion complexes [89]. FAK is a cytoplasmic tyrosine kinase located in focal adhesion complexes. It regulates integrin-mediated cell spreading, migration, and signaling events [129]. Fg in the tumor microenvironment regulates expression of genes involved in cell cycle regulation and metabolism, promotes tumor growth, and limits tumor cell senescence [89]. Analogous to its role as a major adhesive glycoprotein involved in the final stages of blood clotting, Fg deposition, along with other adhesive glycoprotein, potentially provides a matrix that serves as a scaffold for adherence with other proteins or receptors on cells to mitigate cellular responses.

It has been shown that Fg interacts with several cell types through cell-specific integrins and other receptors [130]. Since Fg is predominantly found in the blood, it naturally interacts first with blood cells and ECs. Only after crossing the EC layer in brain microvessels, it becomes possible for Fg to interact with astrocytes and then with neurons. Fg has been shown to interact with the integrin α_IIb_β_3_ on platelets and mast cells resulting in platelet aggregation, thrombus formation, and affects systemic blood pressure regulation [130]. Fg’s interaction with α_M_β_2_ on microglia and macrophages causes their activation, infiltration, cytokine release, and phagocytic activity [130].

## 5. Conclusions

In this work we have discussed important aspects of the biosynthesis and the physiological and pathological effects of Fg, its chains, its family proteins, and its derivatives. The present review clarifies some discrepancies arising from referring interchangeably to effects of Fg and fibrin and to implications of Fg chains in consequences of effects of Fg protein. Various disorders associated with the different blood levels of Fg, its derivatives, and the fibrinogen-like proteins with emphasis on their effects were discussed. It can be concluded that the higher levels of Fg that are associated with inflammation have a variety of effects resulting in many pathological complications. A role of HFg in memory reduction during neuroinflammatory diseases such as TBI and AD is an example. In some cases, high levels of Fg can cause inflammatory responses, e.g., microvascular permeability. Low levels of Fg, as one of the key components of blood coagulation and thrombogenesis, mainly result in enhanced bleeding during severe trauma and some genetic disorders. We also presented some signaling mechanisms involved in Fg effects during various diseases. Some of the significant effects of Fg/fibrin are summarized in Table (Table 1). Further work needs to be carried out to fully understand functional effects of Fg, its chains, and derivatives during various pathologies, mainly focusing on neurodegeneration and cancer problems.

## Figures and Tables

**Table 1:** Effects of fibrinogen/fibrin, fibrinogen chains, and fibrinogen-like proteins 1 and 2 during various diseases.

Protein/Chain	Disease	Protein Level or Condition	Role in Pathology and/or Outcome	References
Fg	Colon cancer	↑	Biomarker. Low survival.	[4]
Fg	Gastric cancer	↑	Biomarker. ↑ lymph node and liver metastasis, ↓ clinical outcome.	[73]
Fg	Cervical cancer	↑	Biomarker. Low survival.	[74]
Fg	Renal cell carcinoma	↑	Biomarker. Low survival.	[75]
Fg	Hepatocellular carcinoma	↑	Biomarker. Low survival.	[76]
Fg and D-dimer	Breast cancer	↑, ↑	Biomarker. Accelerated tumor growth and low survival.	[81]
Fg and D-dimer	DIC	↓, ↑	Biomarker. Low survival.	[57]
Fg	RA	↑	Biomarker. Hypercoagulation and inflammation.	[6]
Fg	SLE	↑	Biomarker. ↑ Atherothrombosis.	[7]
Fg	TBI	↑	↑ cerebrovascular permeability.	[11,96]
Fg	TBI	↑	Extravascular deposition of Fg, ↑ neuronal death, ↓ STM.	[98]
Fg	TBI	↑	↑ extravascular formation of Fg/fibrin containing protein complexes.	[9,11,96]
Fg	AD	↑	↑ perivascular formation of Fg/fibrin containing protein complexes	[131]
Fg	AD	↑	↑ risk of AD and dementia	[8,132]
Fg	COPD	↑	Biomarker. ↑ risk of death.	[65]
FGA precursor protein	AD	↑ in CSF	Biomarker. Mild cognitive impairment and dementia.	[66]
FGA precursor protein	Liver cancer	↑ in CSF	Biomarker. ↑ survival rate.	[78]
FGA	Human lung adenocarcinoma	↑	Cell apoptosis, inhibits tumor growth and metastasis.	[79]
FGB	Polymorphism of *FGB* promoter	↑	↑ plasma Fg. ↑ risk of atherothrombosis.	[43,60]
FGB	In patients undergoing coronary artery bypass grafting	*FGB*-C148T polymorphism	Results in preoperative HFg and postoperative ↑ CRP, ↑ IL-6.	[61]
FGG	Hereditary hypofibrinogenemia with hepatic storage	Mutation location in exons 8 and 9 of the *FGG* gene	Protein aggregation in the endoplasmic reticulum, liver diseases of variable severity.	[49]
FGL1	Acute inflammation	↑	Acute phase reactant	[38,39]
FGL1	Cancer (in general)	↑	Immunosuppressor through binding to LAG3. Poor prognosis.	[118]
FGL2	HBV orHCV	↑	Correlates with viral loading, degree of liver inflammation and disease severity.	[40,87]
FGL2	HBV orHCV	↑	Immunosuppressive activities	[120]
FGL2	Autoimmune glomerulonephritis	↓	Autoimmune glomerulonephritis with age	[121]
FpB	Inflammation	↑	↑ chemotaxis of PMN and fibroblast.	[83]

## Abbreviations

AD	Alzheimer’s disease
CSF	Cerebrospinal fluid
COPD	Chronic Obstructive Pulmonary Disease
CRP	C-reactive protein
DIC	Disseminated intravascular coagulation
FG	Fibrinogen
FGA	Fg alpha chain
FGB	Fg beta chain
FGG	Fg gamma chain
FGL1	Fg-like protein 1
FGL2	Fg-like protein 2
FpB	Fibrinopeptide B
HBV	Hepatitis B virus
HCV	Hepatitis C virus
HFg	Hyperfibrinogenemia
IL-6	Interleukin-6
LAG3	Lymphocyte-activation gene
MS	Multiple sclerosis
PMN	Polymorphonuclear neutrophils
RA	Rheumatoid arthritis
STM	short-term memory
SLE	Systemic lupus erythematosus
TBI	Traumatic brain injury
